# Hepatocyte Nuclear Factor 4α (HNF4α) Is a Transcription Factor of Vertebrate Fatty Acyl Desaturase Gene as Identified in Marine Teleost *Siganus canaliculatus*

**DOI:** 10.1371/journal.pone.0160361

**Published:** 2016-07-29

**Authors:** Yewei Dong, Shuqi Wang, Junliang Chen, Qinghao Zhang, Yang Liu, Cuihong You, Óscar Monroig, Douglas R. Tocher, Yuanyou Li

**Affiliations:** 1 Marine Biology Institute & Guangdong Provincial Key Laboratory of Marine Biotechnology, Shantou University, Shantou, 515063, Guangdong, China; 2 Institute of Aquaculture, School of Natural Sciences, University of Stirling, Stirling, FK94LA, Scotland, United Kingdom; Universiti Sains Malaysia, MALAYSIA

## Abstract

Rabbitfish *Siganus canaliculatus* was the first marine teleost demonstrated to have the capability of biosynthesizing long-chain polyunsaturated fatty acids (LC-PUFA) from C_18_ precursors, and to possess a Δ4 fatty acyl desaturase (Δ4 Fad) which was the first report in vertebrates, and is a good model for studying the regulatory mechanisms of LC-PUFA biosynthesis in teleosts. In order to understand regulatory mechanisms of transcription of Δ4 *Fad*, the gene promoter was cloned and characterized in the present study. An upstream sequence of 1859 bp from the initiation codon ATG was cloned as the promoter candidate. On the basis of bioinformatic analysis, several binding sites of transcription factors (TF) including GATA binding protein 2 (GATA-2), CCAAT enhancer binding protein (C/EBP), nuclear factor 1 (NF-1), nuclear factor Y (NF-Y), hepatocyte nuclear factor 4α (HNF4α) and sterol regulatory element (SRE), were identified in the promoter by site-directed mutation and functional assays. HNF4α and NF-1 were confirmed to interact with the core promoter of Δ4 *Fad* by gel shift assay and mass spectrometry. Moreover, over-expression of HNF4α increased promoter activity in HEK 293T cells and mRNA level of Δ4 *Fad* in rabbitfish primary hepatocytes, respectively. The results indicated that HNF4α is a TF of rabbitfish Δ4 *Fad*. To our knowledge, this is the first report on promoter structure of a Δ4 *Fad*, and also the first demonstration of HNF4α as a TF of vertebrate *Fad* gene involved in transcription regulation of LC-PUFA biosynthesis.

## Introduction

Long-chain polyunsaturated fatty acids (LC-PUFA) play a pivotal role in many biological processes such as the regulation of gene expression, endocytosis/exocytosis, membrane fluidity, ion-channel modulation, biosynthesis of eicosanoids and other autacoids [1–3]. As key enzymes in the pathway of LC-PUFA biosynthesis, fatty acyl desaturases (Fad) convert a single bond between two carbon atoms (C-C) to a double bond (C = C) within a fatty acyl chain [4]. In mammals, the Fad with Δ5 activity is encoded by the *FADS1* gene, while Δ6 activity is encoded by the *FADS2* gene [5]. However, to date, all teleost Fads have been shown to be *Fads2* genes, albeit they have subfunctionalised to carry out several desaturation functions, including Δ5, Δ4 and bifunctional Δ6/Δ5 activities as well as the Δ6 desaturation as in mammals and other vertebrates [6].

The Δ4 Fad is responsible for converting adrenic acid (22:4n-6) to n-6 docosapentaenoic acid (22:5n-6) and n-3 docosapentaenoic acid (DPA; 22:5n-3) to docosahexaenoic acid (DHA; 22:6n-3), respectively. It was previously found in lower eukaryotes including protozoans (*Trypanosoma cruzi*, *Trypanosoma brucei*, *Leishmania major*) [7] and microalgae (*Thraustochytrium* sp, *Euglena gracilis*, *Pavlova lutheria*, *Isochrysis galbana*, *Pavlova viridis*, *Thalassiosira pseudonana*, *Emiliania huxleyi*) [8–14]. Recently, a Δ4 Fad was found, for the first time in vertebrates, in a marine teleost the rabbitfish *Siganus canaliculatus* by our group [15], and subsequently was reported in several other teleosts including Senegalese sole *Solea senegalensis* [16], Mexican silverside *Chirostoma estor* [17] and striped snakehead *Channa striata* [18]. The discovery of Δ4 Fad in vertebrates enables a more direct mechanism for DHA formation from EPA via DPA in comparison to the Sprecher pathway involving two elongation steps of EPA to 24:5n-3, a Δ6 desaturation to 24:6n-3 and partial β-oxidation to DHA [19] and this has attracted the interest of researchers and it has been implicated in the establishment of balanced PUFA ratios in human embryonic kidney 293 cells [20] and to enhance the production of DHA in Chinese hamster ovary cells [21]. Beyond its use as a biotechnological tool, the presence of Δ4 Fad in some farmed fish species has been suggested as an opportunity to develop species in the aquaculture industry with high LC-PUFA biosynthetic capability that are less dependent on provision of these essential nutrients in the diet [22].

It has been generally accepted that freshwater fish have the capability of LC-PUFA biosynthesis from the C_18_ precursors linoleic acid (LNA; 18:2n-6) and α-linolenic acid (ALA; 18:3n-3), while marine fish have very limited LC-PUFA biosynthetic ability [23]. Accordingly, fish oil rich in LC-PUFA must be added to formulated feeds of marine fish so as to meet the essential fatty acid (EFA) requirements for normal growth and development [24]. This restricts the application of formulated feed and the expansion of the aquaculture industry, due to the ever-increasing demand for the only major sources of n-3 LC-PUFA, the marine ingredients fishmeal and fish oil, that have finite supply, limited availability and increasing cost [22]. However, the discovery in rabbitfish *S*. *canaliculatus* provides an opportunity to address such problems. Our group demonstrated for the first time in marine fish, that the rabbitfish is able to biosynthesize LC-PUFA [25, 26] and furthermore cloned genes encoding all the enzymatic activities of fatty acyl desaturation and elongation required for LC-PUFA biosynthesis from C_18_ PUFA, including Δ4 Fad and Δ6/Δ5 Fad, as well as two elongases of very long-chain fatty acids (Elovl4 and Elovl5) [15, 25, 27].

The particular LC-PUFA biosynthesis characteristics of the rabbitfish, especially its unusual Δ4 desaturation capability, provides an interesting model to investigate the regulatory mechanisms controlling LC-PUFA biosynthesis in fish, which is poorly understood at present. Recently, we demonstrated that miR-17 was involved in the regulation of LC-PUFA biosynthesis in rabbitfish liver by targeting Δ4 *Fad* at a post-transcription level, which was the first report in vertebrates [28]. The molecular mechanism of transcriptional regulation of Δ4 *Fad* is one of the key steps in the clarification of regulatory mechanisms of LC-PUFA biosynthesis in rabbitfish. In the present study, the 5’ upstream region of rabbitfish Δ4 *Fad* was cloned and the core promoter region analyzed using a dual luciferase reporter system. A combination of bioinformatic analysis and site-directed mutagenesis was applied to investigate the presence of important binding sites for transcription factors (TFs) within the promoter region of the Δ4 *Fad* gene. In addition, TFs interacting with the conserved region of the core promoter were further characterized and confirmed by electrophoretic mobility shift assay (EMSA) and liquid chromatography coupled with tandem mass spectrometry (LC-MS). Among them HNF4α was identified, which was the first demonstration of it as a TF of *Fad* gene in vertebrate. The role of HNF4α in transcriptional regulation of the Δ4 *Fad* gene was further confirmed by showing that over-expression of HNF4α in HEK 293T cells and in rabbitfish primary hepatocytes increased promoter activity and mRNA levels of the Δ4 *Fad* gene, respectively. To our knowledge, this is the first report on promoter structure of a Fad-like gene with Δ4 activity, and also the first demonstration of HNF4α involved in transcriptional regulation of Fad in vertebrates.

## Materials and Methods

### Ethics statement

In present study, all procedures performed on fish were in accordance with the National Institutes of Health guide for the care and use of Laboratory animals (NIH Publications No. 8023, revised 1978) and approved by the Institutional Animal Care and Use Committee of Shantou University (Guangdong, China). Rabbitfish were captured from the coast near Nan Ao Marine Biology Station (NAMBS) of Shantou University, Southern China, the field study did not involve endangered or protected species. All surgery was performed under 0.01% 2-phenoxyethanol (Sigma-Aldrich, St. Louis, MO, USA) anesthesia, and all efforts were made to minimize suffering of fish.

### Rabbitfish genomic DNA extraction and cloning of 5’ flanking sequence of Δ4 *Fad*

Genomic DNA was extracted from 25 mg muscle of rabbitfish *S*. *canaliculatus* using the proteinase K and phenol protocol [29]. After the muscle samples were rapidly finely chopped, they were treated with 1ml extraction buffer, incubated at 55°C overnight, and then treated with 1 ml of a mixture solution (phenol/chloroform/isoamyl alcohol, 25:24:1- by vol). After 30 min rocking treatment at room temperature, the organic and aqueous phases was separated by centrifugation at 13250 × g for 5 min, and the upper aqueous phase was transferred into a clean 1.5 ml microfuge tube. An equal volume of isopropanol was added to the tube to precipitate the DNA. After centrifugation at 13250 × g for 15 min at 4°C, the isopropanol was removed, the nucleic acid pellet was rinsed with 1 ml 70% ethanol and centrifuged at 13250 × g for 5 min, and the ethanol removed. Finally, the nucleic acid pellet was dried in air for 15 min and dissolved in 0.1 ml TE buffer (pH 8.0) by rocking gently overnight at 4°C, and then stored at -20°C before use.

Previously, we cloned the full-length cDNA of rabbitfish Δ4 *Fad* which contained an open reading frame of 1335 bp encoding a protein with 445 amino acids [15]. According to the blast result of querying the *Danio rerio* genome DNA (National Center of Biotechnology Information, NCBI) with the rabbitfish Δ4 *Fad* gene, and according to the manufacturer’s protocol for the Genome Walking Kit (TaKaRa, Dalian, China), three primers SP1, SP2, SP3 (Table 1) were designed to amplify genomic sequence of rabbitfish Δ4 *Fad*, which was considered as the known sequence. A thermal asymmetric interlaced (TAIL) PCR was performed with rabbitfish genomic DNA as template, while the primer AP4 in the Genome Walking Kit and three specific antisense primers SP4, SP5, SP6 designed from the known sequence, were used to clone the 5’ flanking sequence of the Δ4 *Fad* (Table 1). After three rounds of PCR, a fragment of upstream sequence was recovered and isolated by gel extraction, inserted into pMD18-T Vector (TaKaRa, Dalian, China), and sequenced (Sangon Biotech Co., Ltd, China). The sequencing results showed the existence of two non-coding exons of the Δ4 *Fad* in the 5’ untranslated region (UTR) sequence, confirming that the fragment obtained by PCR was indeed the 5’ flanking sequence of the Δ4 *Fad*.

**Table 1:** Primers used for 5’ flanking sequence cloning, deletion mutant construction, EMSA, mRNA construction and Q-PCR.

Subject	Primers	Nucleotide sequence
TAIL PCR for 5’ flanking sequence cloning	SP1	5’-GATGTTGACTGTTTAAA-3’
	SP2	5’-TGACCTCCACCTCCCAT-3’
	SP3	5’-TGACCAACCACTGGTC-3’
	SP4	5’-GTGAACCAGGCTTTGTCTGAAGAGTGT-3’
	SP5	5’-CATCCACTGGTCATTCCTGTTGCT-3’
	SP6	5’-ACCAGCATCTGACTTGCAGCCATT-3’
pfu-PCR for deletion mutant construction	DF4	5’-CCCGCTAGCGAACACTCTGCTTCACCTACTT-3’
	DF3	5’-CCCGCTAGCATATAGACATTATAAAGCAACCTCT-3’
	DF2	5’-CCCGCTAGCTTAGTAAAGCCCAAGAAAGG-3’
	DF1	5’-CCCGCTAGCTAATATTTAATTATTCAGTCCACAG-3’
	DR	5’-CCCAAGCTTCATCCTCACTGCTGTCTCTG-3’
EMSA for gel shift	BF (5’biotinlabeled)	5’-GGACTTGGCAACTGCCTCCTTAT-3’
	BR (5’biotinlabeled)	5’-ATATTGGACTTACAAAATCC-3’
	UF (5’ unlabeled)	5’-GGACTTGGCAACTGCCTCCTTAT-3’
	UR (5’ unlabeled)	5’-ATATTGGACTTACAAAATCC-3’
mRNA construction for *HNF4α* overexpression	T7 promoter primer	5’-TAATACGACTCACTATAGGG-3’
	paHNF4α	5’-GAAGGAAAAGGCTTCGGAGGGTTGTTA-3’
Q-PCR Detection for target gene expression	QS-Δ4 Fad	5’-GAACACCATTTGTTCCCGAG-3’
	QA-Δ4 Fad	5’-TTCAGTGCCCTGACGACG-3’
	QS-HNF4α	5’-CCGACTCTACAGAGCATCACCTG-3’
	QA-HNF4α	5’-TCATTAGCAGAACCTCCGAGAAG-3’
	QS-18S rRNA	5’-CGCCGAGAAGACGATCAAAC-3’
	QA-18S rRNA	5’-TGATCCTTCCGCAGGTTCAC-3’

Notes: Restriction sites underlined are *Nhe*I (GCTAGC) and *Hin*dIII (AAGCTT) in expression vector pGL4.10.

### Reporter vector construction of progressive deletion mutants for identification of 5’ flanking sequence of Δ4 *Fad*

**Fig 1:**
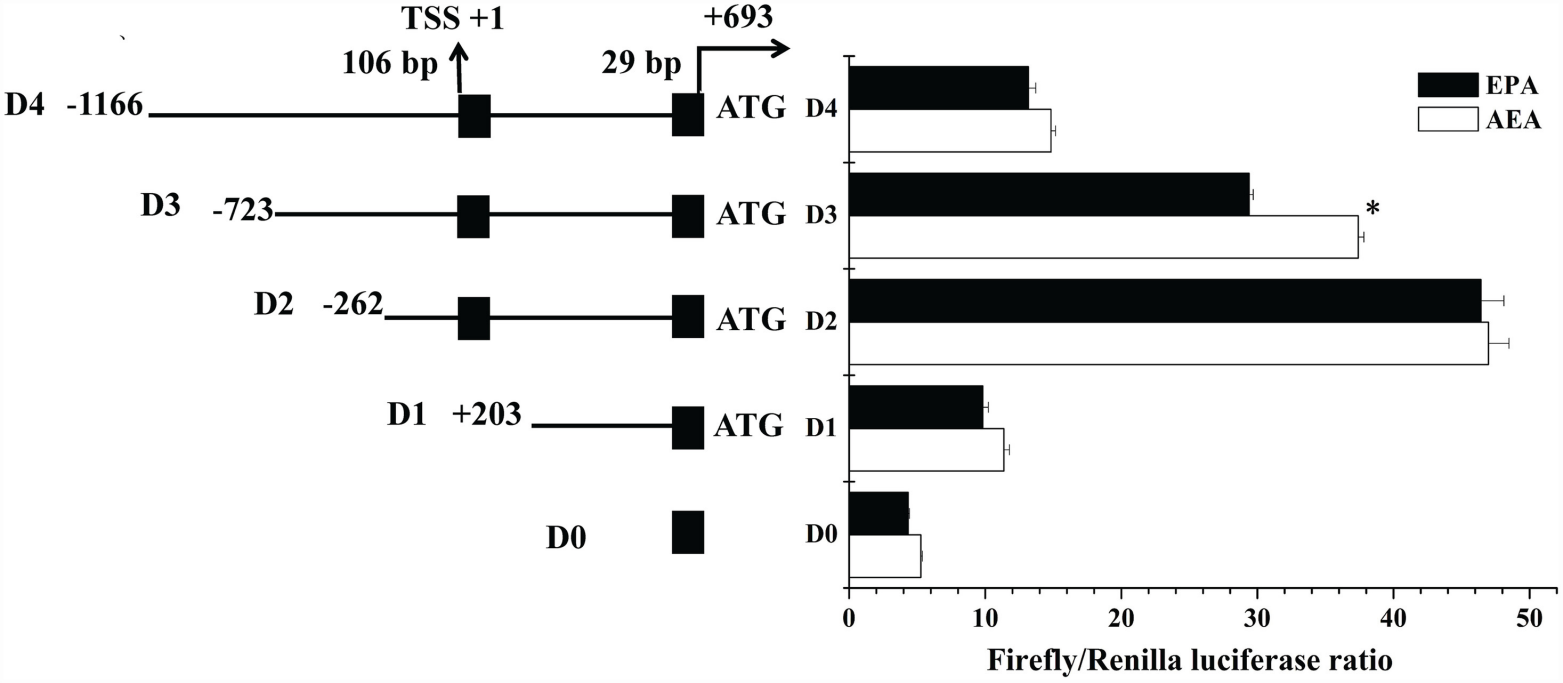
The structure and deletion analysis of 5’ flanking sequence of *Siganus canaliculatus* Δ4 *Fad*. Deletion constructs are represented on the left. Non-coding exons are shown by black boxes. The intron is indicated with a black line between the two exons. Sequence is numbered relative to the transcription start site (TSS), speculated to be the first base of the first 5’ non-coding exon. Promoter activity of constructs is represented with the values representing normalized activity (Firefly luciferase: Renilla luciferase) on the right. Asterisks indicate that the influence of eicosapentaenoic acid (EPA), compared with absolute ethyl alcohol (AEA) treatment, was significant (Student’s *t*—test; *P*<0.05).

For identifying the core promoter region within the cloned 5’ flanking sequence of the rabbitfish Δ4 *Fad*, PCR using one of the forward primers (DF4, DF3, DF2, DF1), all containing a 5’ *Nhe*I site, and the antisense primer DR containing a *Hin*dIII site, were performed to obtain the full-length promoter fragment (D4: 1862 bp) and three deletion mutant fragments (D3, 1419 bp; D2, 958 bp; D1, 493 bp) (Fig 1). The PCR reaction was carried out using 2 × *Pfu* PCR Master Mix (Tiangen Biotech, Beijing, China) and genomic DNA as template. PCR products were digested by restriction endonuclease *Nhe*I and *Hin*dIII (New England Bio labs, Ipswich, MA, UK) and inserted into similarly restricted pGL4.10 [luc2] vector (Promega). The distance of insert fragments D4, D3, D2 and D1 to the putative transcription start site (TSS) +1, predicted as the first base of the first non-coding exon, was -1166 bp, -723 bp, -262 bp and +203 bp, respectively (Fig 1). The constructs were verified for accuracy by sequencing (Shanghai Sangon Biotech Co). Recombinant plasmids of insert fragments (D4, D3, D2 and D1) and pGL4.10 were isolated with High Pure Plasmid Isolation Kit (Roche, Mannheim, Germany) for further transfection in human embryonic kidney (HEK293T) cells (Chinese Type Culture Collection, Shanghai, China).

### Prediction of TF binding sites in 5’ flanking sequence of Δ4 *Fad*

In order to identify possible TF binding sites in the core promoter of the rabbitfish Δ4 *Fad*, online software TRANSFAC^®^ and TF Binding^®^ were used to search the potential binding sites in the 5’ flanking sequence of the Δ4 *Fad*. Since no genome data or promoter structure of Δ4 *Fad* is currently available in any vertebrate, and all teleost *Fads* including Δ6 *Fad*, Δ6/Δ5 *Fad*, Δ4 *Fad* belong to *FADS2* gene cluster [6], an alignment of the core promoter regions between rabbitfish Δ4 *Fad* and the reported promoter sequences of Δ6 desaturase from *Dicentrarchus labrax* [30], *Gadus morhua* [31], *Salmon salar* [31], *Homo sapiens* [32] and Δ6/Δ5 desaturase from *Danio rerio* genome (*D*. *rerio* strain Tuebingen chromosome 25 genomic scaffold, Zv9 scaffold3372) was performed to search for conserved *cis* elements (BioEdit v7.0.9, Tom Hall, Department of Microbiology, North Carolina State University, USA).

### Reporter vector construction of site-directed mutants for identification of the predicted TF binding sites in the core promoter of rabbitfish Δ4 *Fad*

To determine the potential functions of predicted TF binding sites on the core promoter activity, site-directed mutation of recombinant plasmids was carried out. For the rabbitfish Δ4 *Fad* promoter, the construct of deletion mutant D2 was treated as wild-type and site-directed mutants were produced from this with mutation sites designed in the middle of the primer according to the manufacturer's protocol. The strategy of site directed mutation was shown in Table 2 and the mutation was performed with the Muta-direct^TM^ site-directed mutagenesis kit (SBS Genetech, Shanghai, China). PCR with recombinant plasmids pGL 4.10-D2 as template was performed by one cycle (95°C for 30 sec), 15 cycles (95°C for 30 sec, 55°C for 1 min, 72°C for 5 min 12 sec). After the reaction, the PCR product was incubated on ice for 5 min, and digested with Mutazyme^TM^ (SBS Genetech, Shanghai, China) for 1 h. Finally, the digested product was transformed into DH5α competent cells for selection, and positive clones subsequently sequenced (Shanghai Sangon Biotech Co.). Site-directed mutation of insert fragment D2 in recombinant plasmids was confirmed by sequence comparison with the wild-type deletion mutant D2. The site-directed mutation plasmids from D2 were named as SD1, SD2, SD3, SD4, SD5, SD6, SD7 referring to the binding sites of the corresponding TFs GATA binding protein 2(GATA-2), CCAAT enhancer binding protein (C/EBP), nuclear factor 1 (NF-1), TATA box binding protein (TBP), nuclear factor Y (NF-Y), sterol regulatory element (SRE) and hepatocyte nuclear factor 4α (HNF4α) in the core promoter region, respectively. These site-directed mutants were isolated with High Pure Plasmid Isolation Kit for further transfection in HEK 293T cells.

**Table 2:** The TF binding sites predicted by software online and information for site-directed mutants.

TF	Software	Position	Predicted site	Mutation site
GATA-2	TF binding^®^	-225	TAGGATATCT	GA→CT
C/EBP	TF binding^®^	-194	ACTTTGCAAGAAAA	TG→CT
NF-1	TF binding^®^	-151	ACTTGGCAACTGCCTCCT	TG→GT
TBP	TRANSFAC^®^	-123	TTTATTG	TTTATTG→×
NF-Y	comparison	-73	CGCGCCGATTGG	CGCGCCGATTGG→×
SRE	comparison	-41	CTCGAATGATCGGCTCGGAATTT	CTCGAATGATCGGCTCGGAATTT→×
HNF4α	TRANSFAC^®^	+104	TTTGTAAGTCCAATAT	AAGTCCAATA→×

Notes: The position of each element is numbered relative to the presumed TSS. The bases underlined are the mutation site for site-directed mutant (bases replacement or deletion).“×” means deletion.TF binding^®^, TRANSFAC^®^ are two main software online for these predicted TFs.

### Cell culture and transfection for detection of reporter vector activity

In order to detect the influence of the reporter vectors above (progressive deletion mutants and site-directed mutants) on promoter activity, the mutants were transfected into the mammalian cell line, HEK 293T cells, which were seeded in 96-well cell culture plates in 100 μl High Glucose Dulbecco's Modified Eagle Medium (DMEM) (Gluta MAX) (Gibco, Life Technologies, USA) with 10% fetal bovine serum per well (FBS, Sijiqing Biological Engineering Material Company, Hangzhou, China). To confirm the core promoter regions sufficient to initiate transcription and the area responsive to PUFA, progressive deletion mutants of Δ4 *Fad* promoter were transfected into HEK 293T cells grown in the presence of eicosapentaenoic acid (EPA, Cayman Chemical Co., Ann Arbor, USA) or absolute ethyl alcohol (AEA) carrier alone. HEK 293T cells were grown for 24 h to 80% confluence, then transfected with 100 ng of each reporter firefly luciferase construct with Lipofectamine^®^ 2000 Reagent (Invitrogen, Carlsbad, CA, USA), and co-transfected with 0.01 ng of vector pGL4.75 (Promega Corporation), an internal control vector to normalize variations in transfection efficiency, constitutively expressing renilla luciferase by CMV promoter. The empty vector pGL4.10 with no promoter sequence in the 5’ flanking region of the reporter gene (firefly luciferase) was used as a negative control in each transfection assay. The LC-PUFA, EPA, had been demonstrated to been effective suppresser of lipogenesis in HEK293 [33] and AS cells [31], and thus it was chosen to search for a PUFA response region along the *Δ4 Fad* promoter. Based on these earlier studies on effects of PUFA, 50 μM EPA was an appropriate concentration for detection. Fresh cell culture medium with 50 μM EPA or with the same volume absolute ethyl alcohol (control) was replaced at 24 h after transfection.

The effect of site-directed mutation on the transcriptional activity of the core promoter was determined by co-transfecting HEK 293T cells with site-directed mutants and pGL4.75. The ratio of site-directed mutants and pGL4.75 in the transfection complex was the same as for the progressive deletion mutants above. The cells were incubated in 100 μl DMEM +10% FBS and transfected with plasmid complex. Each plasmid complex was transfected in triplicate in three independent experiments. Cell culture medium was replaced with 75 μl DMEM +10% FBS at 24h after transfection. Luciferase assays were performed at 48 h after transfection with the Dual-Glo^TM^ luciferase assay system (Promega), and chemical luminescence intensity was detected in duplicate readings using a microplate reader (Infinite M200 Pro, Tecan, Switzerland). The promoter activity was calculated from the chemical luminescence intensity ratio of firefly: renilla luciferase for each construct, and then compared with the activity of vector pGL4.10 luciferase [31].

### Electrophoretic Mobility Shift Assay (EMSA)

To confirm the existence of TF binding sites in the core promoter of rabbitfish Δ4 *Fad*, nuclear and cytoplasmic proteins were extracted from rabbitfish liver tissue by Beyotime Nuclear Extract Kit (Beyotime Institute of Biotechnology, Haimen, China) and quantified by Sangon non-Interference Protein Assay Kit (Sangon, Shanghai, China). A 5’ end biotin-labeled probe of 289 bp covering the transcription elements was designed and incubated with the proteins so as to determine whether interaction existed between the TFs and the core promoter of the Δ4 *Fad*. The 5’ end biotin-labeled probe was obtained from 50 μl PCR reaction system including 20 μl ddH_2_O, 25 μl 2 × *Pfu* PCR Master Mix, 2 μl 10 mM 5’ end biotin-labeled forward primer, 2 μl 10 mM 5’ end biotin-labeled reverse primer and 1 μl pGL 4.10-D2 recombinant plasmid (100 ng/μl), while the competitor probe was made from the same system with unlabeled primers. PCRs consisted of an initial step (95°C for 3 min), followed by 35 cycles (95°C for 30 sec, 51°C for 30 sec, 72°C for 40 sec) and a final extension step (72°C for 5 min). Both the labeled and unlabeled primers for probes in EMSA were supplied by Shanghai Sangon Biotech Co., Ltd. According to the manufacturer’s instructions, EMSA of 20 μl reaction system was performed with Beyotime Chemiluminescent EMSA Kit (Beyotime Institute of Biotechnology, Haimen, China) in the following groups: negative control group (15 μl ddH_2_O, 4 μl 5 × binding buffer, 0 μl proteins, 1 μl containing 36 ng labeled probe), experimental groups of liver cytoplasmic proteins (13 μl ddH_2_O, 4 μl 5 × binding buffer, 2 μl containing 9.3 μg liver cytoplasmic proteins, 1 μl containing 36 ng labeled probe) and liver nuclear proteins (13 μl ddH_2_O, 4 μl 5 × binding buffer, 2 μl containing 9.3 μg liver nucleus proteins, 1 μl containing 36 ng labeled probe), competitor groups of liver cytoplasmic proteins (13 μl ddH_2_O, 4 μl 5 × binding buffer, 2 μl containing 9.3 μg liver cytoplasmic proteins, 10.47 μl containing 3600 ng unlabeled probe, 1 μl containing 36 ng labeled probe) and liver nucleus proteins (13 μl ddH_2_O, 4 μl 5 × binding buffer, 2 μl containing 9.3 μg liver nucleus proteins, 10.47 μl containing 3600 ng unlabeled probe, 1 μl containing 36 ng labeled probe). The binding reaction was subjected to a 4% non-denaturing polyacrylamide gel and transferred to a nylon membrane. The 5’ end biotin-labeled DNA was detected using a streptavidin-horseradish peroxidase conjugate and a chemiluminescent substrate. The signal was then detected by autoradiography with X-OMAT BT X-ray film (Kodak, USA).

### Isolation and identification of TFs binding to core promoter by LC-MS

To identify the binding of TFs to core promoter, Pure Proteome^TM^ Streptavidin Magnetic Beads (Millipore, Bedford, MA, USA) and Amicon^®^Ultra-0.5 Centrifugal Filter Devices (Millipore, Bedford, MA, USA) were used to bind and isolate proteins, respectively. The DNA-protein complex of the gel shift band was obtained from 100 μl EMSA reaction system including 20 μl liver nucleus protein (105.6 μg), 10 μl biotin-labelled probe (360 ng), 20 μl binding buffer and 50 μl H_2_O. One hundred μl of magnetic bead suspension was put into a 1.5 ml micro centrifuge tube, and a magnetic stand used to collect beads and the storage buffer was removed. After pretreating the magnetic beads by washing using 500 μl Tris-buffered saline with Tween^®^ 20 surfactant (TBST) for 10 s, 100 μl biotinylated protein was added to the magnetic beads and incubated with gentle mixing for 60 min at room temperature. The magnetic stand was applied to collect the beads, and bound protein eluted using TBST buffer. 500 μl sample was added to the Amicon^®^ Ultra filter (Millipore, Bedford, MA, USA) and centrifuged at 14,000 × g for 10 min. The isolated protein solution was analyzed by liquid chromatography coupled with tandem LC-MS technology (Beijing Genomics Institute, China).

After adjusting the pH to 8.5 with 1 M ammonium bicarbonate, total protein (100 μg) extracted from each sample was chemically reduced for 1 h at 60°C by adding DTT to 10 mM and carboxyamidomethylated in 55 mM iodoacetamide for 45 min at room temperature in the dark. Then Trypsin Gold (Promega) was added to a final substrate/enzyme ratio of 30:1 (w/w). The trypsin digest was incubated at 37°C for 16 h. After digestion, the peptide mixture was acidified by 10 μl of formic acid for further MS analysis. After protein digestion, each peptide sample was desalted using a Strata X column (Phenomenex), vacuum-dried and then resuspended in a 200 μl volume of buffer A (2% ACN, 0.1% FA). After centrifugation at 20000 × g for 10 min, the supernatant was recovered to obtain a peptide solution with a final concentration of approximately 0.5 μg/μl. 10 μl supernatant was loaded on a LC-20AD nano-HPLC (Shimadzu, Kyoto, Japan) by the autosampler onto a 2cm C18 trap column. The peptides were eluted onto a 10 cm analytical C18 column (inner diameter 75 μm) packed in-house. The samples were loaded at 8 μL/min for 4 min, then the 44 min gradient was run at 300 nL/min starting from 2 to 35% B (98% ACN, 0.1% FA), followed by 2 min linear gradient to 80%, and maintenance at 80% B for 4 min, and finally return to 5% in 1 min. The peptides were subjected to nanoelectrospray ionization followed by tandem mass spectrometry (MS/MS) in a QEXACTIVE (ThermoFisher Scientific, San Jose, CA) coupled online to the HPLC. Intact peptides were detected in the Orbitrap at a resolution of 70 000. Peptides were selected for MS/MS using high-energy collision dissociation (HCD) operating mode with a normalized collision energy setting of 27.0 and ion fragments were detected in the Orbitrap at a resolution of 17500. A data-dependent procedure that alternated between one MS scan followed by 15 MS/MS scans was applied for the 15 most abundant precursor ions above a threshold ion count of 20000 in the MS survey scan with a following Dynamic Exclusion duration of 15 s. The electrospray voltage applied was 1.6 kV. Automatic gain control (AGC) was used to optimize the spectra generated by the Orbitrap. The AGC target for full MS was 3e6 and 1e5 for MS2. For MS scans, the m/z scan range was 350 to 2000 Da. For MS2 scans, the m/z scan range was 100–1800 Da. Functional annotations of the proteins were conducted using Blast2GO program against the non-redundant protein database.

### Construction of HNF4α over-expression vector and verification of its function on Δ4 *Fad* promoter activity

To verify the effect of rabbitfish HNF4α on Δ4 *Fad* promoter activity, over-expression vector pcDNA3.1-HNF4α was constructed with clone vector plasmid pEASY-Blunt-Zero-HNF4α (GenBank: JF502073.1) and expression vector pcDNA3.1 (Invitrogen) double digested by *Xba*I and *EcoR*I-HF (New England Bio labs, Ipswich, UK), then with target HNF4α fragments and empty vector pcDNA3.1 ligated by T4 DNA ligase. Constructed vector pcDNA3.1-HNF4α was sequenced (Shanghai Sangon Biotech Co.) for accuracy. Progressive deletion mutants of Δ4 *Fad* promoter (100 ng/well) and pcDNA3.1-HNF4α (50 ng/well) were co-transfected into HEK293T cell lines seeded in 96-well cell culture plates, while pGL4.75 (0.02 ng/well) was added as an internal control vector. HEK 293T cell was incubated in 100 μl DMEM + 10% FBS and transfected with plasmid complex. Each plasmid complex was transfected in triplicate in three independent experiments. Cell culture medium was replaced with 75 μl DMEM + 10% FBS at 24 h after transfection. Luciferase assays were performed at 48 h after transfection with the Dual-Glo^TM^ luciferase assay system, and chemical luminescence intensity was detected in duplicate readings using a microplate reader.

### In vitro mRNA transcription of rabbitfish HNF4α

mRNA transcription was performed on a linearized DNA template containing T7 promoter and rabbitfish HNF4α cDNA sequence (GenBank: JF502073.1) using mMESSAGE mMACHINE^®^ T7 Ultra Kit (Ambion, Austin, TX) according to the manufacturer to generate capped mRNA with a poly(A) tail. Plasmid pEASY-Blunt-Zero-HNF4α was constructed with pEASY-Blunt-Zero vector (TRANS Gen, China) and HNF4α cDNA, which was used as a template for a linearized DNA template above with T7 promoter primer and antisense primer of HNF4α containing termination codon in a pfu-PCR reaction (Table 1). PCR procedure were one cycle (94°C for 3 min), 30 cycles (94°C for 30 sec, 65°C for 30 sec, 72°C for 1 min 30 sec), one cycle (72°C for 10 min). Finally, the product containing HNF4α mRNA was purified with MEGAclear^TM^ Kit (Ambion, Austin, TX), and stored in -80°C for further transfection into rabbitfish primary hepatocytes, described previously [28]

### Rabbitfish HNF4α mRNA transfection into the primary hepatocytes and Q-PCR detection for the influence to Δ4 *Fad* expression

The rabbitfish primary hepatocytes were seeded in two 6-well plates (Eppendorf, Hamburg, Germany) in DMEM/F12 with 15% FBS medium. When grown for 48 h to 80% confluence, the cells were then transfected with 5 μg/well HNF4α mRNA by Lipofectamine^TM^ MessengerMAX^TM^ Reagent (Invitrogen). At 48 h after transfection, the primary cells transfected or not were treated with Trypsin-EDTA (Invitrogen), and centrifuged 1500 × g, 2 min to precipitate the cells. The total RNA was extracted from the cell pellet by RNeasy^®^ Plus Mini kit (QIAGEN, USA), detected by electrophoresis and quantified by Nanodrop 2000 Spectrophotometer (Thermo Fisher Scientific, Wilmington, USA). Then 0.2 μg total RNA were reverse transcribed into cDNA with oligo-dT primer by the SuperScript^®^ III First-Strand Synthesis System (Invitrogen). Expression of target genes (*HNF4α*, *Δ4 Fad*) was measured by Q-PCR. Copy numbers of the genes *HNF4α*, *Δ4 Fad* (GenBank: GU594278) were normalized to reference gene *18S rRNA* (GenBank: AB276993) calculated by the comparative threshold cycle (Ct) method [34]. Specific primers for target genes are shown in Table 1. The PCR was carried out on a Lightcycler 480 system (Roche, Basel, Switzerland) in a final volume of 20 μl, which contained 10 μl of SYBR Green Super mix (Bio-rad, Hercules, CA, USA), 1 μl of specific primer (10 μM), 6 μl ddH_2_O and 2 μl cDNA template (10 ng/μl). The PCR program was consisted of an initial DNA denaturation of 94°C for 5 min, followed by 45 cycles at 95°C for 10 s, annealing 60°C for 20 s, extension 72°C for 20 s, with a final extension step at 95°C for 5 s, 65°C for 1 min, 40°C for 10 s. The reactions of each sample were performed in triplicate.

### Statistical analysis

All data were presented as means ± SEM. The data of promoter activity influenced by progressive deletion, site-directed mutations, pcDNA3.1-HNF4α (n = 3), Q-PCR data of *HNF4α* and Δ4 *Fad* gene expression after rabbitfish HNF4α mRNA treatment (n = 6) were analyzed by one-way analysis of variance (ANOVA) followed by Tukey's multiple comparison test or Student's t-test using Origin 7.0. A significance of *P* < 0.05 was applied to all statistical tests performed.

## Results

### The structure of rabbitfish Δ4 *Fad* gene promoter

Upstream sequence of 1859 bp from the initiation codon ATG was cloned as the candidate for the Δ4 *Fad* promoter. The first base of the first non-coding exon was regarded as the putative TSS, which was defined as the +1 position in the sequence. There was an intron of 561 bp length between two noncoding-exons, the first exon was 106 bp and the second 29 bp. Based on progressive deletion of the 5’flanking sequence of the Δ4 *Fad*, the deletion of fragments D4 (-1166 bp to -724 bp) and D3 (-723 bp to -263 bp) caused a gradual increase of promoter activity, whereas significantly reduced promoter activity occurred when the region of fragment D2 (-262 bp to +203 bp) was deleted, suggesting that the core promoter region was located at -262 bp to +203 bp (Fig 1). Additionally, notable repression of promoter activity by EPA occurred with deletion mutant D3, whereas the other three deletion mutants (D4, D2 and D1) showed no response to EPA, indicating that the PUFA response region was located from -723 bp to -263 bp (Fig 1). Very low promoter activity was detected in the negative control D0 (pGL4.10), (Fig 1).

### Putative TF binding sites in the core promoter of rabbitfish Δ4 *Fad*

**Fig 2:**
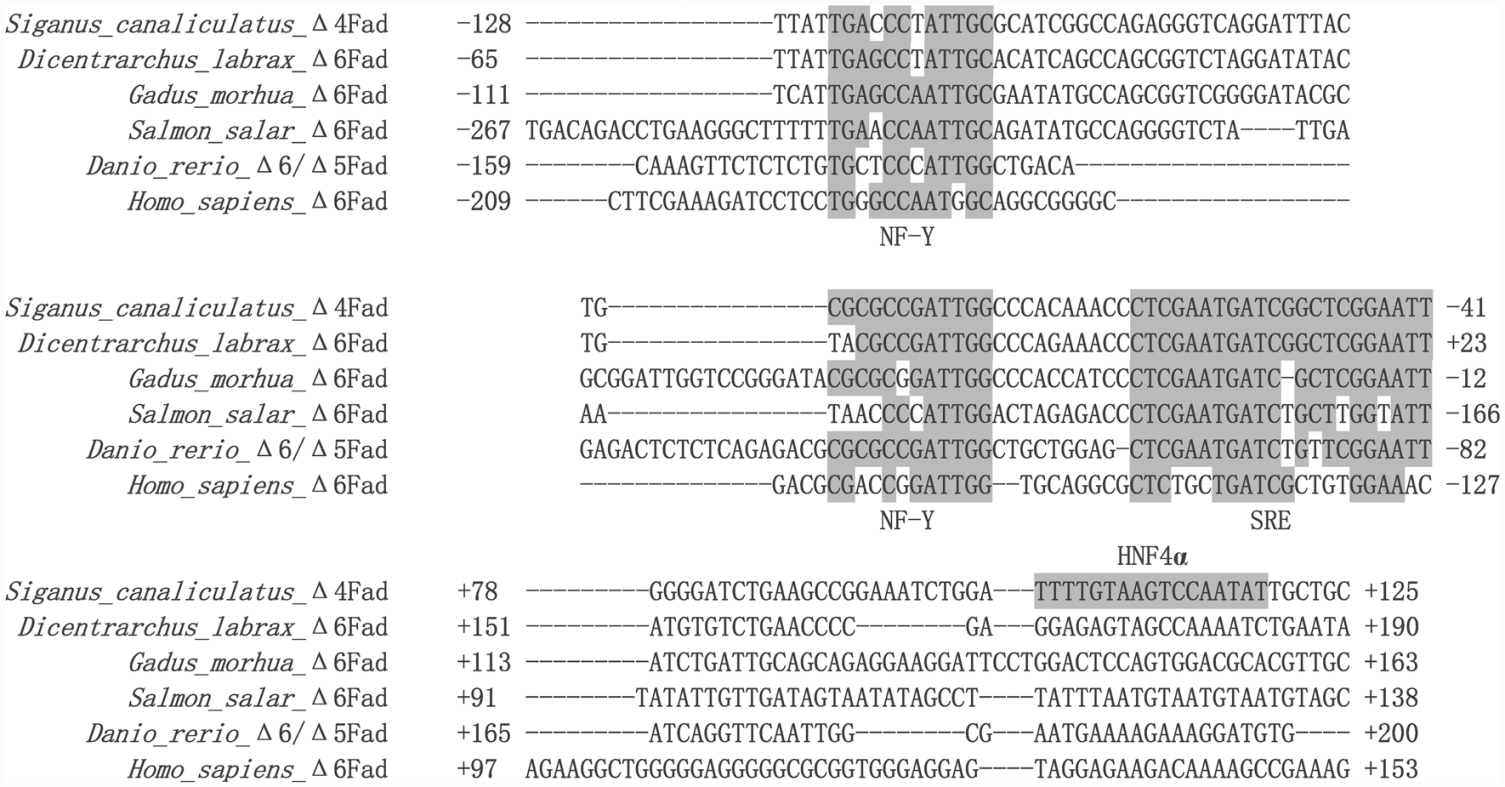
The core promoter regions alignment of *Fads2* from *Siganus canaliculatus*, *Dicentrarchus labrax*, *Gadus morhua*, *Salmon salar*, *Danio rerio* and *Homo sapiens*. While the *S*. *canaliculatus Fads* encodes a Δ4 desaturase, the other Fads2-like genes encode desaturases with Δ6 activity or Δ6/Δ5 activity. The sequences of latter four promoters were obtained from the corresponding reported references and *Danio rerio* Δ6/Δ5 *Fad* promoter was from NCBI genome data. The sequences are all numbered relative to the transcription start site. The conserved elements of NF-Y and SRE are labeled with shaded box.

**Fig 3:**
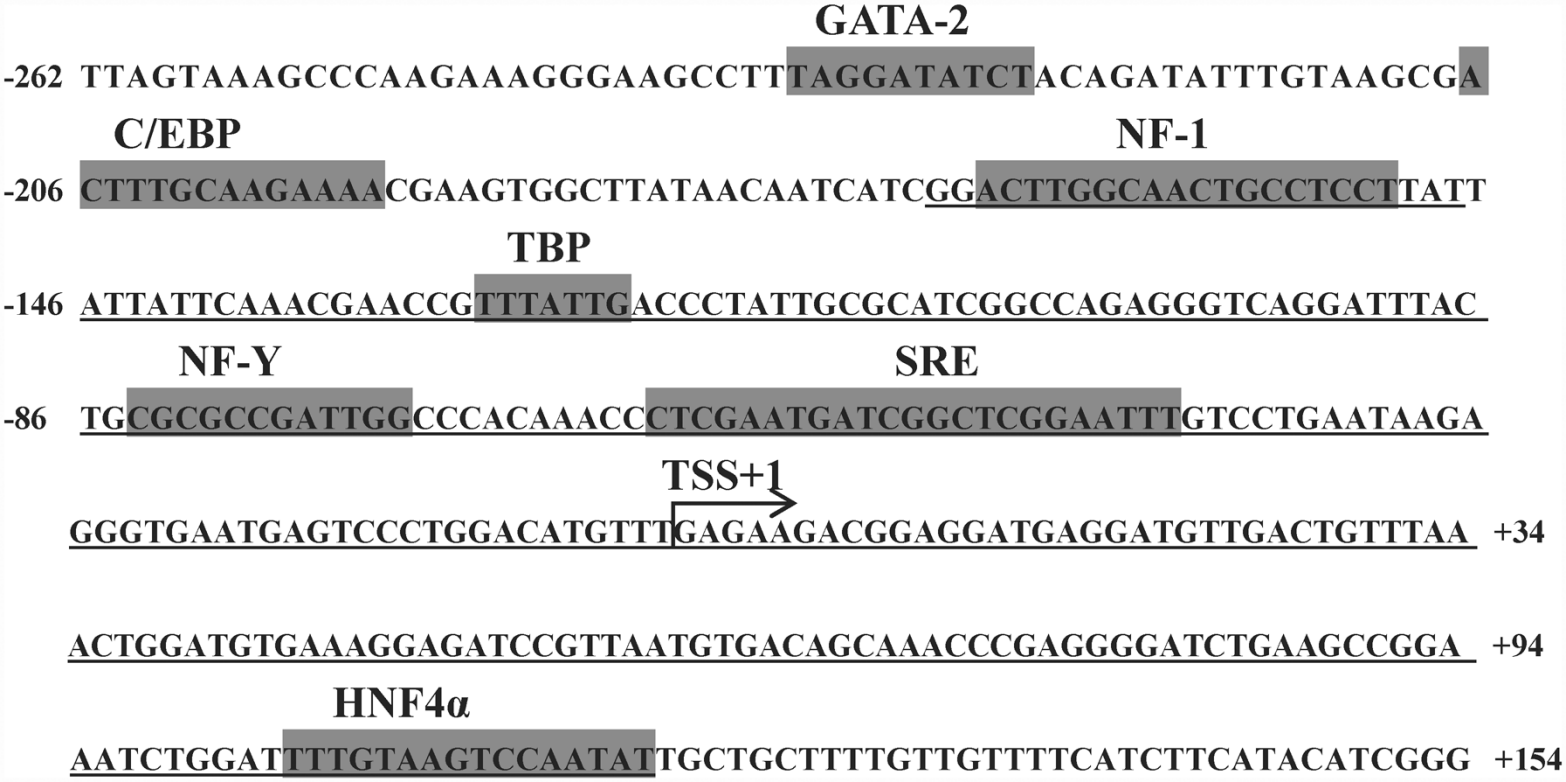
The core promoter region of *Siganus canaliculatus* Δ4 *Fad*. Numbers are relative to the supposed transcription start site (TSS). The TF binding sites predicted by corresponding software are shown above with shadow parts in the sequence. The underlined parts indicate the probe region of EMSA.

Using bioinformatics software including TRANSFAC^®^ and TF binding^®^ (Table 2), five TFs including GATA-2, C/EBP, NF-1, TBP and HNF4α were predicted within the core promoter region of rabbitfish Δ4 *Fad*. According to the alignment of the core promoter of rabbitfish Δ4 *Fad* with the reported Δ6 *Fad* promoters of *D*. *labrax*, *G*. *morhua*, *S*. *salar* and *H*. *sapiens* and Δ6/Δ5 *Fad* promoter of *Danio rerio*, two highly conserved elements including NF-Y and SRE were identified in the core promoter region of rabbitfish Δ4 *Fad* (Fig 2). On the basis of the bioinformatic analysis, GATA-2, C/EBP, NF-1, TBP, NF-Y, HNF4α and SRE were identified as potential factors influencing the activity of rabbitfish Δ4 *Fad* promoter (Fig 3). And HNF4α element did not exist in the other *Fad* promoter sequence as observed from the promoter sequence alignment (Fig 2).

### Identification of TF binding sites by site-directed mutagenesis of the core promoter

**Fig 4:**
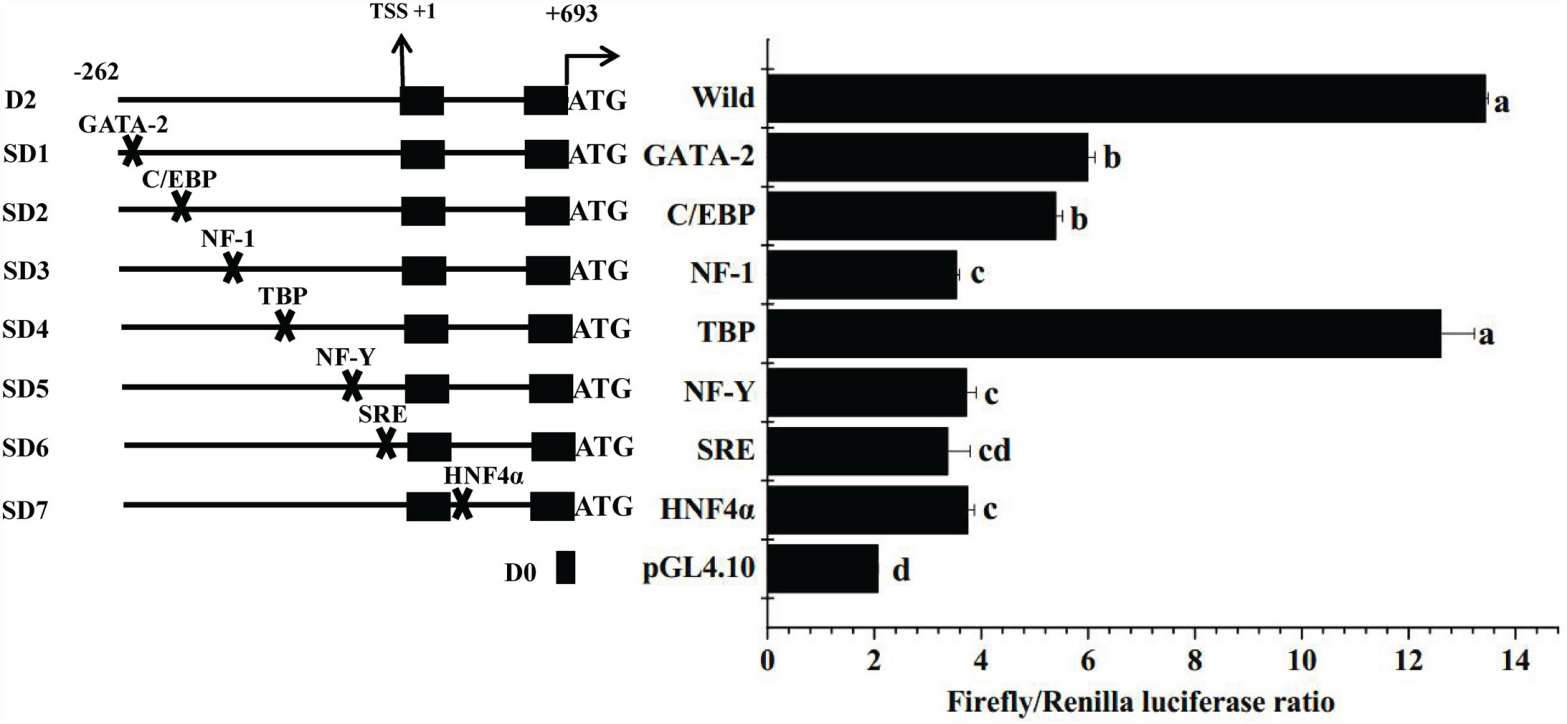
The influence of site-directed mutation on *Siganus canaliculatus* Δ4 *Fad* promoter activity. The site-directed mutants were constructed on the basis of bioinformatics analysis. Each plasmid complex was transfected in triplicate in three independent experiments. Data are means ± SEM (n = 3), bars without share a common letter (a, b, c or d) indicated significant difference among them (ANOVA followed by Tukey's multiple comparison test; *P* < 0.05).

The progressive deletion analysis of 5’ flanking sequence of Δ4 *Fad* using the Dual-Glo^TM^ luciferase assay system confirmed that the core promoter region was located within deletion mutant D2 (-262 to +203). Based on bioinformatic analysis of the core promoter, a series of site-directed mutants of the core promoter was constructed and transfected into HEK 293T cells for detection of transcriptional activity. Compared with the wild type D2, mutation of binding sites for NF-1, NF-Y, HNF4α and SRE caused a highly significant decrease in transcriptional activity, while the effect of GATA-2 and C/EBP mutation was also significant (*P* < 0.05) (Fig 4). Deletion of the TBP element produced no significant difference in promoter activity. pGL4.10 showed very low luciferase activity. The results indicated that the TF binding sites of GATA-2, C/EBP, NF-1, NF-Y, HNF4α and SRE were important for promoter activity.

### EMSA of rabbitfish liver extracted proteins and core promoter

**Fig 5:**
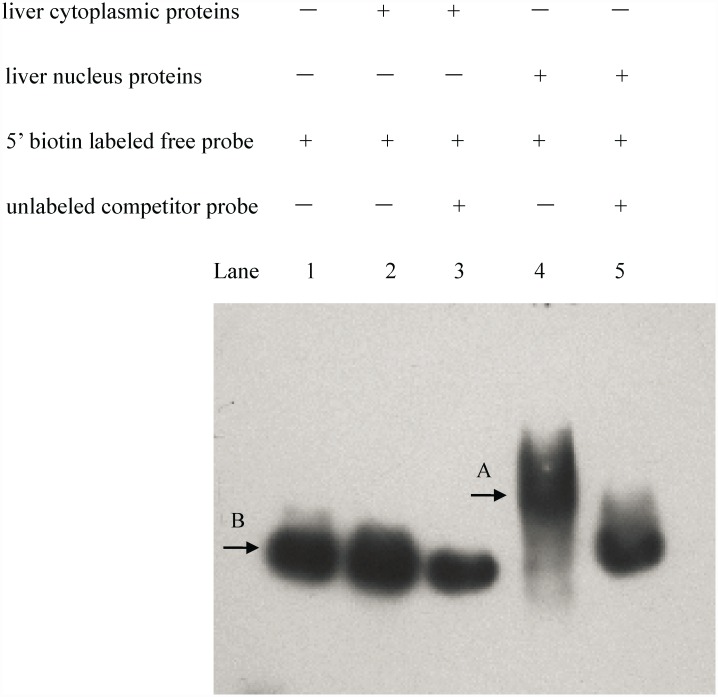
The electrophoretic mobility shift assay (EMSA) of *Siganus canaliculatus* Δ4 *Fad* core promoter with its liver proteins. The reaction was carried out in lane 1 (no proteins, 5’ biotin labeled probe), lane 2 (liver cytoplasmic proteins, 5’ biotin labeled free probe), lane 3 (liver cytoplasmic proteins, unlabeled competitor probe, 5’ biotin labeled free probe), lane 4 (liver nucleus proteins, 5’ biotin labeled probe), lane 5 (liver nucleus proteins, unlabeled competitor probe, 5’ biotin labeled probe). Band A is gel shift of DNA-protein complexes. Band B is the free probe.

To further confirm whether TFs in rabbitfish liver bound to the core promoter, EMSA was performed with liver cytoplasmic and nucleus proteins. The results showed that a gel shift band was observed only in lane 4 consisting of liver nucleus proteins and 5’ biotin labeled probe, which indicated the interaction of nucleus proteins with core promoter of rabbitfish Δ4 *Fad* (Fig 5). No other treatments showed band shifts. The results suggested there should be nucleus proteins binding to the core promoter of rabbitfish Δ4 *Fad*.

### LC-MS analysis of nucleus proteins binding to the core promoter

**Fig 6:**
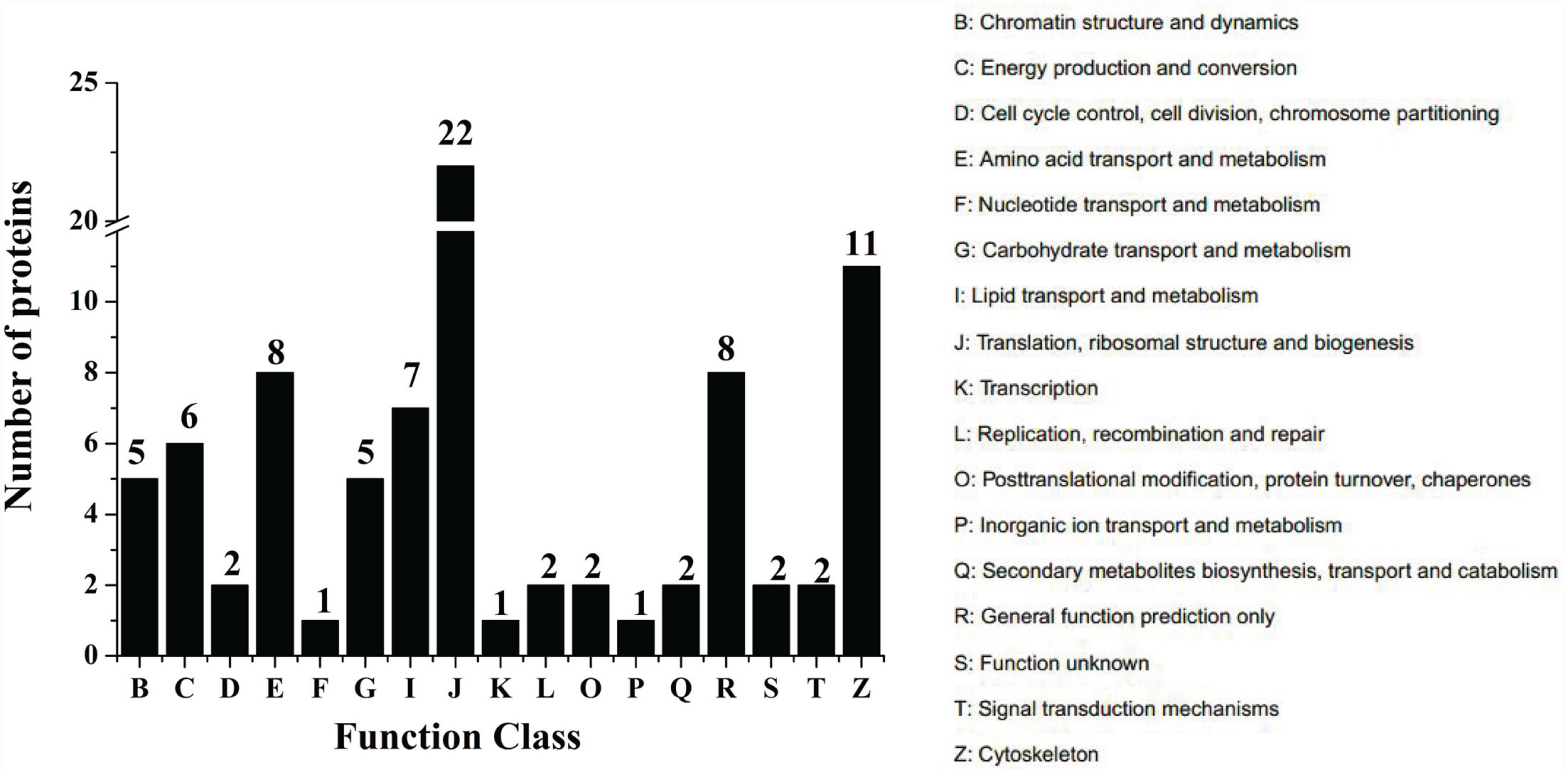
COG Function Classification of nucleus proteins binding to the core promoter. The DNA-protein complex isolated from lane 4 was analyzed by LC-MS. The 83 proteins identified in liver nucleus protein sample were classified into different functions with COG (Cluster of Orthologous Group of proteins) method. The number above the column stands for the amount of proteins identified in COG Function Classification.

In order to confirm the TFs in nucleus proteins binding to the core promoter, the DNA-protein complex isolated from lane 4 was analyzed by LC-MS. The protein sample was digested by trypsin. The database of IPI_Zebrafish (40470 seqs) (ftp://ftp.ebi.ac.uk/pub/databases/IPI) was used to analyze the peptide fragments with the method of GO (Gene Ontology) and COG (Cluster of Orthologous Group of proteins) (Fig 6). There were 294 identified spectra in a total of 14992 spectra, and 83 identified proteins from a total of 141 identified peptides. The identified protein fragments of HNF4α and NF-1 are shown in Table 3. These results provided evidence for the interaction of TFs (HNF4α and NF-1) with the core promoter of rabbitfish Δ4 *Fad*.

**Table 3:** Identified TFs and the corresponding protein fragments.

TF	Protein fragments
HNF4α	YQVQVSLEDYINDR
NF-1	LDLVMVILFK

### The influence of HNF4α over-expression on Δ4 *Fad* promoter activity

**Fig 7:**
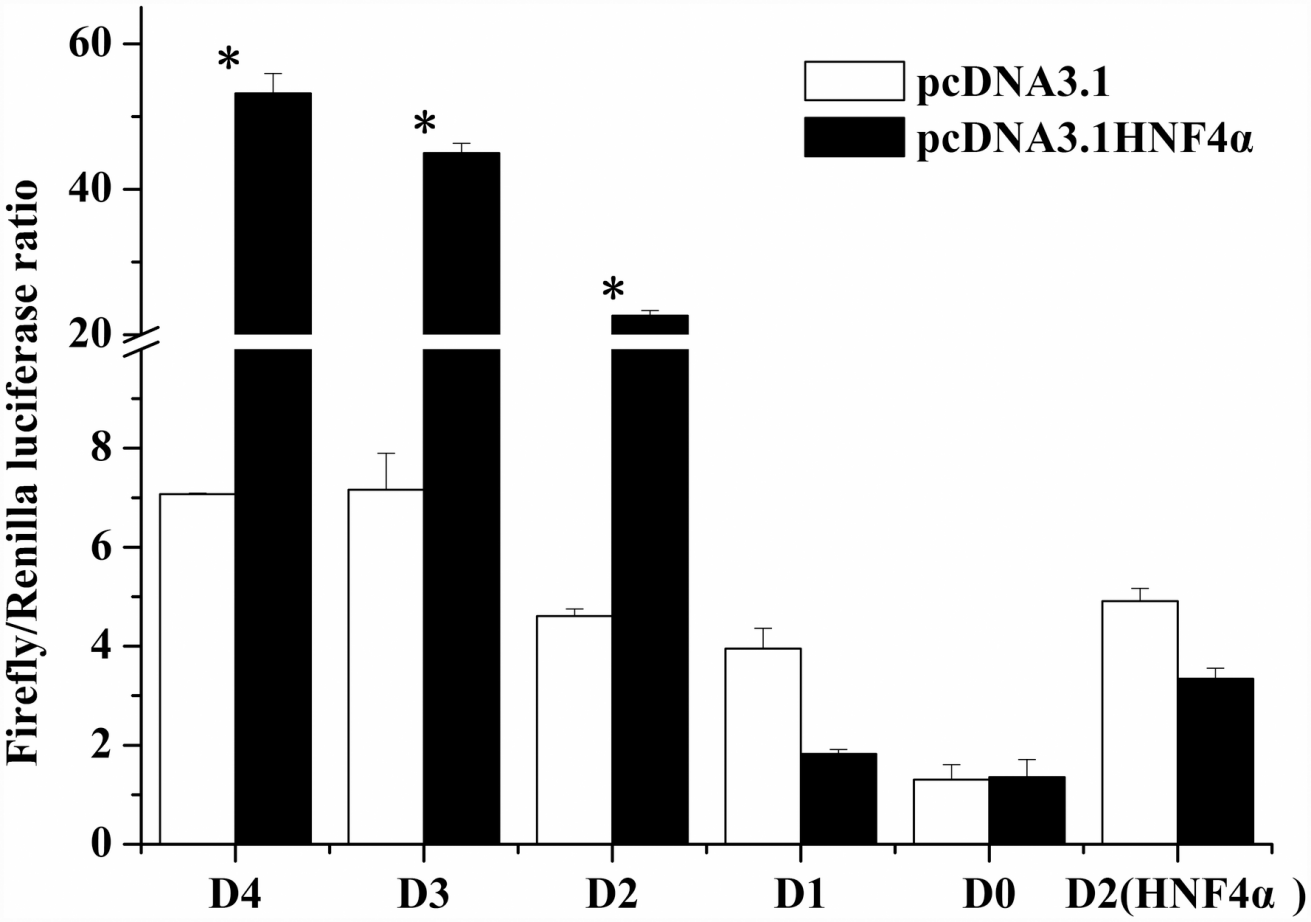
Effects of *S*. *canaliculatus* HNF4α over-expression on activity of Δ4 *Fad* promoter progressive deletion mutants. Each deletion mutant was co-transfected with over-expression vector pcDNA3.1-HNF4α, compared with the control group transfected with empty vector pcDNA3.1. The negative control D0 (pGL4.10) was an empty vector with no promoter sequence upstream the reporter gene. Each plasmid complex was transfected in triplicate in three independent experiments. Y-axis is the F/R luciferase ratio, x-axis stands for different deletion mutants. Asterisks indicate that the influence of HNF4α over-expression on Δ4 *Fad* promoter activity was significant compared with the corresponding control group transfected with empty vector pcDNA3.1 (Student’s *t* -test; *P* < 0.05).

To further confirm the interaction of HNF4α with Δ4 *Fad*, the influence of rabbitfish HNF4α over-expression on Δ4 *Fad* transcription was determined. The over-expression vector pcDNA3.1-HNF4α was constructed and co-transfected into HEK 293T cells with progressive deletion mutants of *Δ4 Fad*. Deletion mutants D1, negative control D0 and site-directed mutant of HNF4α in D2 showed no response to HNF4α treatment, while the activity of D4, D3, D2 was increased significantly by HNF4α over-expression (*P* < 0.05) (Fig 7). These results suggested that HNF4α may improve Δ4 *Fad* expression at a transcriptional level.

### Overexpression of HNF4α increased the transcription of Δ4 *Fad* gene

**Fig 8:**
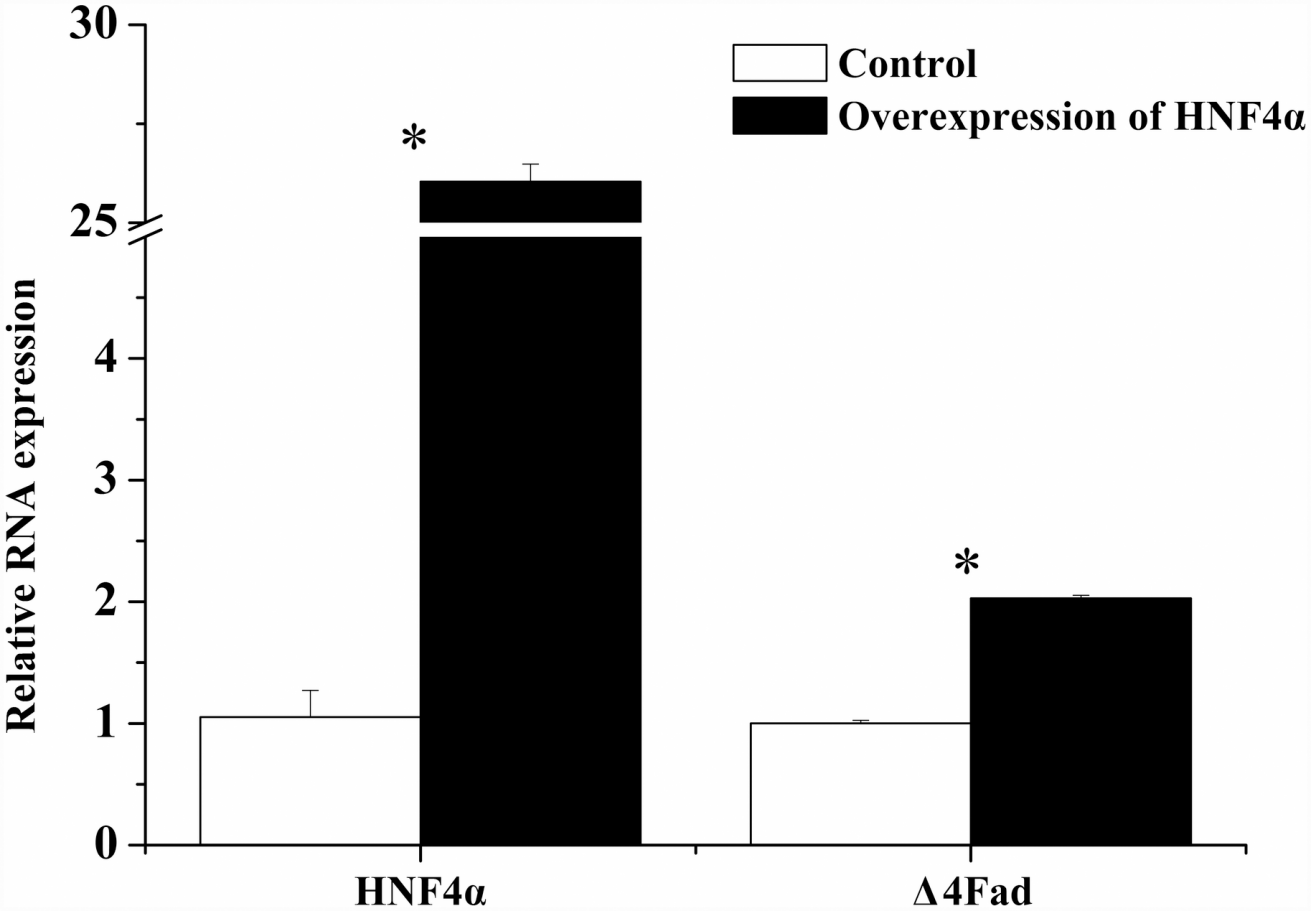
Q-PCR analysis of HNF4α and Δ4 *Fad* gene expression in rabbitfish primary hepatocytes transfected with HNF4α mRNA or not. Relative expression of target genes were quantified for each transcript and were normalized with *18S rRNA* by 2^− ΔΔCt^ method. The white column is the control groups and the black is experiment groups transfected with HNF4α mRNA. Results are means ± SEM (n = 6), the Asterisks indicate that compared with the control group, the influence of HNF4α mRNA treatment to HNF4α and Δ4 *Fad* gene expression is significant in experiment group (Student’s *t* -test; *P* < 0.05).

Besides the above results in HEK 293T cells, the function of HNF4α on Δ4 *Fad* was further confirmed in rabbitfish primary hepatocytes. After the rabbitfish HNF4α mRNA, which was synthesized *in vitro*, was transfected into the rabbitfish primary hepatocytes, the mRNA of Δ4 *Fad* gene was significantly increased (Fig 8). The results suggested that HNF4α may be involved in the regulation of LC-PUFA biosynthesis by targeting Δ4 *Fad* gene.

## Discussion

In the present study, we characterized the structure of *S*. *canaliculatus* Δ4 *Fad* gene promoter. Compared with the previously reported Δ6 *Fad* promoters from *D*. *labrax* [30], *G*. *morhua* [31], *S*. *salar* [31] *H*. *sapiens* [32] and Δ6/Δ5 *Fad* promoter from *Danio rerio* genome, the candidate sequence for *S*. *canaliculatus* Δ4 *Fad* promoter had distinctive features in terms of the core promoter region and TF binding sites, including the identification of HNF4α in vertebrate Fad-like gene transcription.

The position of *S*. *canaliculatus* Δ4 *Fad* core promoter was -262 bp to TSS, while those of Δ6 *Fad* promoters of *D*. *labrax*, *G*. *morhua*, *S*. *salar* and *H*. *sapiens* were -194, -167, -546 and -385 bp, respectively. According to the alignment with the above sequences, the highly conserved NF-Y and SRE elements in vertebrates were also identified in the rabbitfish Δ4 *Fad* promoter. In addition to the NF-Y and SRE conserved elements, there were several different TF binding sites in the Δ6 *Fad* core promoters of the different species. One C/EBPα element of *G*. *morhua* Δ6 *Fad* promoter was discovered in position -99 bp to TSS [31]. For the Atlantic salmon Δ6 *Fad* promoter [31], a Sp1 element was discovered in position -314bp to TSS. In human *FADS2* (Δ6 desaturase), five Sp1 elements and a DR-1 element (PPARα binding element) were predicted in the promoter region [32]. The present study confirmed that binding sites of GATA-2, C/EBP, NF-1, NF-Y, HNF4α and SRE influenced the transcription of *S*. *canaliculatus* Δ4 *Fad*. Therefore, the *S*. *canaliculatus* Δ4 *Fad* promoter shared the same binding site for C/EBP as in *G*. *morhua* Δ6 *Fad* promoter, but did not contain either the Sp1 element that was reported in *S*. *salar* and *H*. *sapiens* Δ6 *Fads2* promoters, or the DR-1 element of the *H*.*sapiens* Δ6 *FADS2* promoter. However, results suggested that the structure of *S*. *canaliculatus* Δ4 *Fad* promoter was highly conserved in comparison to promoters of Δ6 *Fad* or Δ6/Δ5 *Fad* from other fish. This was consistent with all these genes being Fad orthologues [6], regardless of their functionalities as Δ4, Δ6/Δ5 or Δ6 desaturases. However, the HNF4α element in *S*. *canaliculatus* Δ4 *Fad* core promoter, which was predicted by software TRANSFAC^®^, did not exist in the corresponding region of promoter sequences of Δ6 *Fad* from *D*. *labrax* [30], *G*. *morhua* [31], *S*. *salar* [31], and *H*. *sapiens* [32] and Δ6/Δ5 *Fad* from *D*. *rerio* (Tuebingen chromosome 25 genomic scaffold, Zv9_scaffold3372). It is not known at present whether HNF4α was not a common TF involved in Fad regulation as identified herein for rabbitfish, and more *Fad* promoter structure is yet required, specifically from the fish with Δ4 *Fad* including Senegalese sole [16], Mexican silverside [17] and striped snakehead [18]. To date, there are no other reports on the regulatory role of HNF4α in Fad transcription.

Mutation of the GATA-2 and C/EBP binding sites had significant influences on *S*. *canaliculatus* Δ4 *Fad* transcription, in consideration of the probe length (less than 300 bp), the probe of core promoter for the EMSA did not contain these two elements, and so these factors were not further studied for interaction between nucleus proteins and core promoter. With respect to EMSA, the LC-MS protein analysis did not identify TFs NF-Y and SREBPs in the core promoter, which might be caused by low expression of NF-Y and SREBPs in the *S*. *canaliculatus* liver sample as a low content of these TFs would reduce the opportunity to bind to the probe of rabbitfish Δ4 *Fad* core promoter. Although LC-MS did not identify the existence of GATA-2, C/EBP, NF-Y or SREBPs, according to the site-directed mutation assay, these TFs might still be the potential regulator in the transcription of *S*. *canaliculatus* Δ4 *Fad*.

HNF4α and NF-1 were confirmed as regulatory factors of transcription of *S*. *canaliculatus* Δ4 *Fad*. HNF4α is a TF of the nuclear receptor (NR) superfamily that constitutively binds fatty acids [35, 36], the binding of PUFA including ALA (18:3n-3), EPA (20:5n-3) and DHA (22:6n-3) to HNF4α represses its influence on activating transcription [37]. Furthermore, HNF4α influences the transcription with the formation of homodimer [38]. Up to now, there are more than 1000 genes targeted by HNF4α in human liver [39], involving functions such as detoxification, bile acid metabolism, lipoprotein metabolism/secretion, carbohydrate metabolism, lipogenesis, hormones, urea cycle and alcohol metabolism [39, 40]. With respect to lipid and cholesterol metabolism, HNF4α is important for the activation of various target genes such as ApoCIII, Cyp7α hydroxylase [41, 42], fatty acid synthase [40] and stearoyl-CoA desaturase [39]. However, little is known about the regulation of HNF4α on Fad transcription. A previous study on the regulation of human *FADS2* (Δ6 desaturase) transcription found that HNF4α did not affect Δ6 desaturase expression when co-transfecting CV1 cells with expression vectors of human *FADS2* promoter and HNF4α [32]. The present study predicted an HNF4α element in rabbitfish Δ4 *Fad* promoter. Mutation of this site caused a significant reduction of Δ4 *Fad* promoter activity. Moreover, the proteins identified by LC-MS confirmed the existence of HNF4α in the core promoter. All these results confirmed the existence of a binding site for HNF4α in the *S*. *canaliculatus* Δ4 *Fad* promoter. Finally, the over-expression of *S*. *canaliculatus* HNF4α also showed that this TF could increase the process of Δ4 *Fad* transcription in HEK 293T cells. Promoter activity of D1, D0 and HNF4α element mutant showed no major difference between treatment with empty pcDNA3.1 vector and pcDNA3.1-HNF4α. While compared with the control group (pcDNA3.1 vector), D4, D3 and D2 promoter activity increased significantly after HNF4α overexpression, suggesting HNF4α could increase Δ4 *Fad* promoter activity in HEK 293T cells. To further functionally confirm whether HNF4α is a transcription factor of rabbitfish Δ4 *Fad* gene, the effects of HNF4α overexpression on Δ4 *Fad* mRNA was detected by Q-PCR in rabbitfish primary hepatocytes. HNF4α mRNA overexpression increased Δ4 *Fad* gene expression, suggesting that Δ4 *Fad* could be a target gene of HNF4α. As for NF-1, it activates transcription through direct interaction with basal TFs [43] in the human stearoyl-CoA desaturase gene promoter [44]. In present research, the mutation of NF-1 element in the core promoter also caused a significant reduction of Δ4 *Fad* promoter activity and it was also identified by LC-MS. A further identification of such TFs involved in Δ4 *Fad* gene expression is required to perform as the HNF4α assay above.

In summary, the Δ4 *Fad* promoter of *S*. *canaliculatus* was cloned and characterized in the present study, this representing the first report on the promoter structure of a Δ4 *Fad*, and also the first demonstration of HNF4α as a TF of a vertebrate *Fad* gene, suggesting a new regulatory mechanism in LC-PUFA biosynthesis.
